# The Role of LIM Kinase in the Male Urogenital System

**DOI:** 10.3390/cells11010078

**Published:** 2021-12-28

**Authors:** Juhyun Park, Soo Woong Kim, Min Chul Cho

**Affiliations:** 1 Department of Urology, Asan Medical Center, University of Ulsan College of Medicine, Seoul 05505, Korea; urojpark@amc.seoul.kr; 2 Department of Urology, Seoul National University Hospital, Seoul National University College of Medicine, Seoul 03080, Korea; swkim@snu.ac.kr; 3 Department of Urology, SMG-SNU Boramae Medical Center, Seoul National University College of Medicine, Seoul 07061, Korea

**Keywords:** LIM kinase, male, urogenital system

## Abstract

The LIM kinases (LIMK1 and LIMK2), known as downstream effectors, and the Rho-associated protein kinase (ROCK), a regulator of actin dynamics, have effects on a diverse set of cellular functions. The LIM kinases are involved in the function of the male urogenital system by smooth muscle contraction via phosphorylation of cofilin and subsequent actin cytoskeleton reorganization. Although LIMK1 and LIMK2 share sequence similarities as serine protein kinases, different tissue distribution patterns and distinct localization during cell cycle progression suggest other biological functions for each kinase. During meiosis and mitosis, the LIMK1/2–cofilin signaling facilitates the orchestrated chromatin remodeling between gametogenesis and the actin cytoskeleton. A splicing variant of the LIMK2 transcript was expressed only in the testis. Moreover, positive signals with LIMK2-specific antibodies were detected mainly in the nucleus of the differentiated stages of germ cells, such as spermatocytes and early round spermatids. LIMK2 plays a vital role in proper spermatogenesis, such as meiotic processes of spermatogenesis after puberty. On the other hand, the literature evidence revealed that a reduction in LIMK1 expression enhanced the inhibitory effects of a ROCK inhibitor on the smooth muscle contraction of the human prostate. LIMK1 may have a role in urethral obstruction and bladder outlet obstruction in men with benign prostatic hyperplasia. Moreover, LIMK1 expression was reduced in urethral stricture. The reduced LIMK1 expression caused the impaired proliferation and migration of urethral fibroblasts. In addition, the activated LIMK2–cofilin pathway contributes to cavernosal fibrosis after cavernosal nerve injury. Recent evidence demonstrated that short-term inhibition of LIMK2 from the immediate post-injury period prevented cavernosal fibrosis and improved erectile function in a rat model of cavernosal nerve injury. Furthermore, chronic inhibition of the LIMK2–cofilin pathway significantly restrained the cavernosal veno-occlusive dysfunction, the primary pathophysiologic mechanism of post-prostatectomy erectile dysfunction through suppressing fibrosis in the corpus cavernosum. In conclusion, the LIM kinases–cofilin pathway appears to play a role in the function of the male urogenital system through actin cytoskeleton reorganization and contributes to the pathogenesis of several urogenital diseases. Therefore, LIM kinases may be a potential treatment target in urogenital disorder.

## 1. Introduction

**Figure 1:**
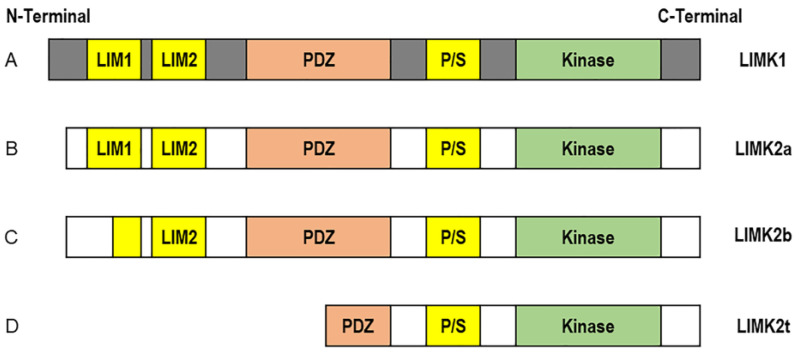
Schematic structure of LIM Kinases. (**A**) The LIMK1 and LIMK2 proteins are composed of an N-terminal kinase domain, two LIM domains, a PDZ domain, proline/serine (P/S)-rich region, and a C-terminal kinase domain. (**B**) LIMK2a, the full-length LIMK2 protein. (**C**) LIMK2b, missing half of the first LIM domain. (**D**) LIMK2t, the testis-specific protein, missing the 2 LIM domains and part of the PDZ domain.

**Figure 2:**
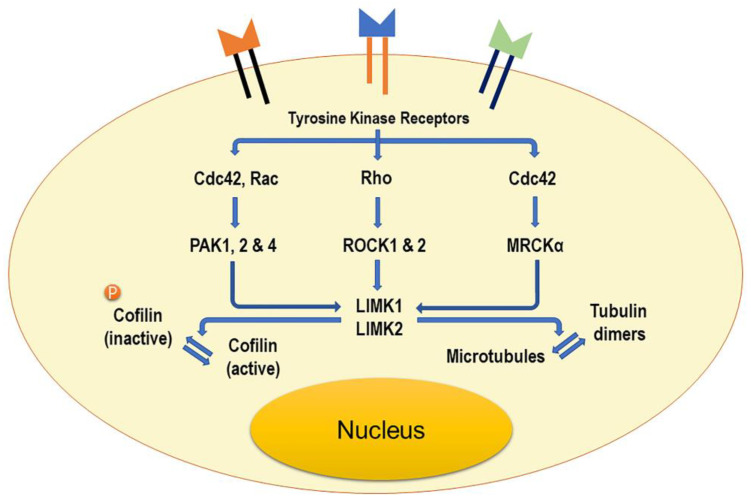
A model for LIMK1 and LIMK2 signaling pathways in normal cells. Several molecules are complicatedly linked and control LIMK activity.

The LIM kinase family consists of two members: LIMK1 and LIMK2. They are composed of an N-terminal kinase domain, two LIM domains, a PDZ domain, proline/serine (P/S)-rich region, and a C-terminal kinase domain. The structure and function of LIMK1 and LIMK2 are very similar because both LIM kinases equally control microtubule dynamics, suggesting that the main difference between these two kinases might be their cell type-specific expression and a different subcellular localization. LIMK2a, LIMK2b, and tLIMK2 are well-known LIMK2 mRNA isoforms [1]. While LIMK2a expresses the full-length transcript as an isoform of LIMK1, LIMK2b lacks half of the first LIM domain, and tLIMK2 is missing both LIM domains and half of the PDZ domain (Figure 1) [2]. Interestingly, the activity of LIMK1 and LIMK2 is regulated in distinctly different ways: LIMK1 is affected by the Rho-family protein, Rac. By contrast, LIMK2 is controlled by Rho and Cdc42, which are mediated by Rho-associated protein kinase (ROCK) and myotonic dystrophy kinase-related Cdc42-binding kinase (MRCKa), respectively [3]. Accordingly, LIMK is an essential component that transduces signals from extracellular stimuli to cytoskeletal networks (Figure 2) [2,3].

Several researchers have long been interested in exploring the ROCK/LIMK/cofilin pathway. ROCK indirectly inhibits the depolymerization of actin filaments. ROCK phosphorylates and activates LIM kinase, which phosphorylates actin-depolymerizing factor (ADF)/cofilin. This sequence results in the stabilization of actin filaments and an increase in their numbers [4]. ROCK/LIMK/cofilin pathway regulates cell morphology, polarity, and cytoskeletal remodeling by regulating actin filaments and cell migration. Studies on the function of the signaling pathway proteins have focused on the cardiovascular system and the central nervous system [5,6,7]. In addition, ROCK and LIM kinases have been suggested as playing a crucial role in tumor cell invasion and metastasis [8,9,10].

In various cancers, including breast, prostate, and colorectal cancers, high expression or activity of each cascade protein is significantly associated with the poor survival rate of patients and aggressive metastasis [8,9,10]. Therefore, blocking the ROCK–LIMK–cofilin pathway can suppress their activities and inhibit tumor cell growth, invasion, and metastasis [8,9,10,11,12,13]. The inhibitors of these signaling pathway proteins might be potential therapeutic agents [4].

So far, several drug-development teams have tried to produce potential substances that control ROCK activity [14]. Fasudil, a potent ROCK inhibitor, prevents cerebral vasospasm after surgery for subarachnoid hemorrhage in China and Japan [15]. Ripasudil, a ROCK inhibitor in the form of eye drops, manages glaucoma and ocular hypertension in Japan [16]. However, the USA and Europe have not yet approved these ROCK inhibitors [15,16]. Because the ROCK inhibitors have potential adverse effects such as systemic vasodilation or hypotension, the selective inhibition of LIM kinase, a downstream effector of ROCK, would be a better choice than direct targeting of ROCK itself in terms of both efficacy and safety [17].

In the past decade, many researchers have revealed the hidden ability of LIM kinase. LIM kinases also control microtubule dynamics, independently of their regulation of actin microfilament [18]. In this review, we focused on the functions and roles of LIM kinase in the male urogenital system. We also investigated the development status of LIMK inhibitors and the applicability of clinical treatment.

## 2. LIM Kinase in the Male Urogenital System and Related Disease

LIMK1 and LIMK2 are encoded by separate genes located on chromosomes 7q11.23 and 22q12.2, respectively [19]. Although the LIM kinases are very homologous, particularly when comparing kinase domains, each LIM kinase may be subject to various regulatory pathways and contribute to distinct and overlapping cellular and developmental functions [19]. LIMK1 has shown remarkably high expression in tissues of the brain, lung, stomach, kidney, and testis. LIMK2 was also observed in most examined tissues, except for kidney glomeruli, testis, and glial cells [2,3].

Normal central nervous system development relies upon the presence of LIMK1, and its deletion has been implicated in the development of the human genetic disorder Williams syndrome [20]. According to recent research, several genes within the 7q11.23 chromosomal region may serve as the causes of Williams syndrome, including ELN, LIMK1, and RFC2 genes [20]. The LIMK1 gene is related to the impaired visuospatial cognition of Williams syndrome. Moreover, the deletion of LIMK2 interferes with normal germ cell development. Researchers found the abnormality of the spermatogenic process in LIMK2-deficient mice [21]. Some portions of seminiferous tubules in the testis of LIMK2-deficient mice contained impaired spermatogenesis and germ cell loss with enhanced apoptosis in spermatocytes [21,22]. LIMK2 might play a role during the meiotic processes of spermatogenesis after puberty [22].

LIMK1 may have a role in urethral obstruction and bladder outlet obstruction in men with benign prostatic hyperplasia (BPH) [23,24,25,26]. LIMK1-dependent actin organization was observed in smooth muscle cells of the prostate, and it has been suggested that LIMK1 is involved in smooth muscle contraction of BPH [25]. In addition, a reduction of LIMK1 expression was noticed in urethral stricture tissues [24].

The accelerated LIMK2–cofilin pathway is associated with cavernosal fibrosis after cavernosal nerve injury [27,28,29,30,31]. Recent evidence demonstrated that chronic inhibition of LIMK2 prevented cavernosal fibrosis and cavernosal veno-occlusive dysfunction (CVOD), the primary mechanism of post-prostatectomy erectile dysfunction.

The major findings of LIM kinase in the male urogenital system are summarized in Table 1.

## 3. LIM Kinase in Gametogenesis

Unlike LIMK1, LIMK2 has at least three functional isoforms generated by alternative splicing with N-terminal sequences [32]. While ordinary transcript LIMK2a presented ubiquitously in various tissues, the LIMK2b was predominantly expressed in the brain [33]. The testis-specific LIMK2 isoform (tLIMK2) was particularly expressed in differentiated, meiotic stages of spermatogenic cells, suggesting its contribution to spermatogenesis [22].

Moreover, tLIMK2 is weakly expressed in the testis at 20 days after birth [22]. Later, the manifestation drastically increases in the postpubertal stage of the testis, while ordinary LIMK2 (LIMK2a) is only observed during the prepubertal stages. Furthermore, tLIMK2 mRNA has been detected in the different stages of spermatogenesis, from spermatocytes to round spermatids. However, tLIMK2 transcripts were not found in spermatogonia and Sertoli cells in adults [22].

Although LIMK2-deficient mice had mainly normal seminiferous tubules containing well-differentiated spermatogenic cells like wild-type testes, some tubules had a few germ cells with reduced diameter [21]. A majority of the abnormal tubules held only a single layer of early spermatocyte stages, and the germ cells of the following stage were reduced in number and were often necrotic. Interestingly, juvenile testis of LIMK2-deficient mice did not show such spermatogenic defects, indicating that impaired spermatogenesis occurred after puberty [34].

Furthermore, the ROCK/LIMK1 pathway regulated Sertoli–germ cell adherens junction dynamics [35]. Sertoli cells had a phagocytotic function that removed apoptotic germ cells during spermatogenesis [36]. Several Rho-families of GTPases affected Sertoli and germ cells in serial order, suggesting that the ROCK/LIMK1 pathway regulated different intracellular functions in spermatogenesis.

Takahashi and his colleagues reported that ectopic expression of LIMK mRNA prevented oocyte maturation by organizing the microtubule-derived precursor of the meiotic spindle [37]. They found that an unusual accumulation of actin at the ectopic expression site disturbed the migration and separation of centrosomes in Xenopus oocytes. Excessive LIMK expression interfered with the proper level of cofilin activity. On the other hand, decreased LIMK during oocyte maturation reduced the phosphorylated cofilin. The enzymatic balance between LIMK and cofilin would be necessary for cytoskeleton dynamics in maturing oocytes and other cellular systems [18]. These studies strongly supported that the ROCK/LIMK/cofilin pathway-mediated cytoskeletal dynamics might be a key component of gametogenesis (spermatogenesis and oogenesis).

## 4. LIM Kinase in Bladder Outlet Obstruction

Obstruction of the prostatic urethra due to increased prostate smooth muscle tone and prostatic enlargement causes BPH and lower urinary tract symptoms (LUTS) [38]. Prostate smooth muscle contraction may be induced by activation of α1-adrenoceptors. Thus, α1-adrenoceptors play a primary role in the etiology and medical treatment of male LUTS. The α1-blockers provoke prostate smooth muscle relaxation and subsequent improvement of urethral and bladder outlet obstruction. Although α1-blockers are considered a primary medical option to improve LUTS/BPH, those medications have limitations due to their insufficient efficacy and stubborn side effects [38,39]. Therefore, researchers have hoped that an accurate understanding of prostate smooth muscle contraction leads to the development of new therapeutic options.

Prostate smooth muscle contraction is linked to numerous intracellular signaling pathways, including several protein kinases and GTPases. P21-activated kinase (PAK) promotes the release of noradrenaline from sympathetic neurons to smooth muscle cells, activating postsynaptic α1-adrenoceptors to promote smooth muscle contraction [40]. Moreover, PAK affects protein kinase C (PKC), ROCK, and related signaling mediators to promote smooth muscle contraction [26]. Several laboratory findings have supported prostate smooth muscle contraction via the suggested pathway. PAK inhibitors, PKC inhibitors, and ROCK inhibitors reduced smooth muscle contractions in a dose-dependent manner [41]. However, these inhibitors are challenging to develop as clinical medications because of unbalanced side effects such as systemic vasodilation or hypotension [17].

As a downstream effector of ROCK, LIMK would be a better choice than directly targeting ROCK itself in both efficacy and safety. LIMK is the critical gateway for regulating smooth muscle contraction through various signaling pathways, including Rho–Rac–Cdc42/ROCK–RAK/LIMK pathway (Figure 2) [41].

Qingfeng Yu et al. reported that the combined application of SR7826 (LIMK1 and ROCK inhibitor) and LIMKi3 (LIMK1/2 inhibitor) inhibited contractions of human prostate tissues. SR7826 and LIMKi3 caused a breakdown of the actin cytoskeleton in prostate smooth muscle cells [25]. A breakdown of this organization reduces smooth muscle contractility and may account for the inhibition of contraction in prostate tissues by SR7826 and LIMKi3. Neither SR7826 nor LIMKi3 affected the myosin light chain (MLC) phosphorylation in prostate tissues, indicating that inhibition of contraction by SR7826 and LIMKi3 was not based on a decrease of MLC phosphorylation. These findings may help to understand the hidden role of LIMK in prostate smooth muscle proliferation and hypertrophy [25]. Thus, LIMK may have a role in prostatic urethral and bladder outlet obstruction in BPH.

## 5. LIM Kinase in Urethral Stricture

Urethral stricture is a narrowing of the urethra lumen by scarring and leads to LUTS [42]. The most common etiologies are idiopathic, traumatic, inflammatory, or iatrogenic [42]. If the pathogenesis of the urethral stricture is not definite, surgical or medical treatment would be unsatisfactory [43]. The common pathological features of urethral stricture include excessive fibroblast proliferation, extracellular matrix deposition, and collagen synthesis in the urethral spongiosum [23,44]. Collagen I and III are the major components of the extracellular matrix. The urethral stricture spongiosum showed a higher proportion of Collagen I to III than the normal urethral spongiosum [44].

RhoA regulates actin filament remodeling in fibroblasts and activates the ROCK-dependent signaling pathway to facilitate scar formation [45,46]. The activation of ROCK for actomyosin-mediated contractility occurs through phosphorylation of LIMK1 and regulatory MLC [10]. Ning Xu et al. demonstrated the effect of local injection of fasudil (ROCK inhibitor) in urethral stricture [24]. Fasudil is known to have antifibrotic effects in various fibrosis diseases [14]. Activation of MLC, LIMK1, and cofilin was detectable in the transforming growth factor (TGF)-β-stimulated urethral fibroblasts. TGF-β can promote the transformation of fibroblasts into myofibroblasts and urethral scar formation [45,46]. Treatment with fasudil (ROCK inhibitor) significantly suppressed fibroblast migration and reduced MLC, LIMK1, and cofilin expression [24]. These findings suggest that the therapeutic potential of fasudil in urethral strictures may partly be due to their inhibitory effect on fibroblast proliferation and collagen synthesis via suppression of the RhoA/ROCK pathways.

Local injection of fasudil could be a choice of application to reduce the concerns of its systemic side effects, but repeated injection treatment is practically tricky [14]. Therefore, new drugs, including LIMK inhibitors, should be developed that prevent ROCK-related scar formation and restore urethral stricture.

## 6. LIM Kinase in Cavernosal Fibrosis

Penile erection is coordinated by psychological, emotional, and hormonal factors. A penile erection requires neurotransmitters, such as nitric oxide (NO) and prostaglandins, released by the cavernous nerve (CN) terminals, which provide parasympathetic innervation to the corpora cavernosa [47]. The CN arises from the pelvic plexus between the bladder and the rectum. Then, the CN runs into the penis along the prostate’s dorsolateral aspect, between the prostate’s capsules [48]. These nerve fibers accompany blood vessels and constitute the neurovascular bundles (NVBs) [49].

Erectile dysfunction (ED) after non-nerve-sparing radical prostatectomy (RP) is an inevitable consequence of the transection of the CN [50]. By contrast, ED after nerve-sparing RP is a complex mechanism and is not entirely understood. Even minimal manipulation of the NVB during RP could cause neuropraxia and affect the recovery of erectile function [51]. A temporary neuropraxia and sacrifice of vascular supply lead to the sustained flaccid penis and insufficient oxygen supply to the penis during the early postoperative period, resulting in the structural change of the corpus cavernosum, such as corporal fibrosis through the TGF-β-mediated pathway [29]. Once structural alterations such as the progressive fibrosis of cavernosal tissues develop, erectile functions are unlikely to recover despite an improvement from temporary neuropraxia. Therefore, CN injury during RP is critical in corporal fibrosis and the development of CVOD [27,28].

Recent studies have demonstrated that the RhoA/ROCK1/LIMK2/cofilin pathway was involved in corporal fibrosis through coordination with TGF-β/sphingosine-1-phosphate signaling after CN injuries [29]. Activation of LIMK2, a downstream mediator of ROCK, leads to cytoskeletal rearrangement through cofilin phosphorylation, promoting fibroblast differentiation into myofibroblast and corporal fibrosis [28]. Nevertheless, short-term inhibition of LIMK2 from the early postoperative period improved cavernosal fibrosis and erectile response to electrostimulation in a CN injury rat model [31]. Moreover, long-term inhibition of the LIMK2/cofilin pathway significantly restored CVOD, the core pathophysiologic mechanism of post-prostatectomy ED, alleviating fibrosis in the corpus cavernosum [27]. Park et al. used the LX7101 to suppress the RhoA/ROCK1/LIMK2/cofilin pathway, the primary pathologic mechanism of cavernosal fibrosis [31]. The LX7101 is a novel class of pyrrolopyrimidine LIMK inhibitors [52]. Recently, a randomized control study of topically administered LX7101 for glaucoma patients has been ongoing [53]. The LX7101 is reported as a dual LIMK2 and ROCK1 inhibitor, whereas the SR7826 mentioned above is a dual LIMK1 and ROCK inhibitor [52]. However, the LX7101 has proved significantly selective for LIMK2 (300-fold compared to ROCK1). Therefore, LX7101 could be a useful option if selective suppression of LIMK2 activity is required [27,31].

The preceding data demonstrated that the short-term and long-term inhibition of LIMK2 improved erectile function but could not completely recover it to normal values [27,31]. In addition, the SM/collagen ratio was not normalized. The apoptosis progression after CN injury, despite chronic LIMK2 inhibition, is a possible explanation for the incomplete recovery of SM/collagen ratio in the cavernosum [30,54]. Thus, future research is needed to explore whether a combination of LIMK2 inhibition with other antiapoptotic agents can restore erectile function to control values.

## 7. Conclusions

The LIMK/cofilin pathway occupies a strategic position in several urogenital diseases. LIMK integrates the signaling mediators, reorganizes the actin cytoskeleton, and contributes to the physiology and pathophysiology of gametocytes, prostate smooth muscles, the urethra, and the corpus cavernosum. Therefore, LIMK may be a potential treatment target in various urogenital disorders. In particular, selective inhibition downstream of ROCK, such as the LIMK/cofilin pathway, would be a better choice for future research. Several LIMK inhibitors are already being tested for the treatment of BPH, urethral stricture, and erectile dysfunction, and these studies are expected to develop into clinical studies in humans soon.

## Figures and Tables

**Table 1:** Summary of major findings of LIM kinase in the male urogenital system.

Male Urogenital System and Related Disease	Major Findings
Testis − infertility	tLIMK2 was mainly expressed in differentiated, meiotic stages of spermatogenic cells, suggesting its contribution to spermatogenesis. In tLIMK2-deficient mice, impaired spermatogenesis occurred after puberty. Ectopic expression of LIMK prevented oocyte maturation by an unusual accumulation of actin that disturbed the migration and separation of centrosomes in Xenopus oocytes.
Prostate − LUTS/BPH	PAK affects PKC, ROCK, and related signaling mediators to promote smooth muscle contraction by activation of α1-adrenoceptors. The combined application of SR7826 (LIMK1 and ROCK inhibitor) and LIMKi3 (LIMK1/2 inhibitor) inhibited contractions of human prostate tissues. SR7826 and LIMKi3 interfered with the LIMK/cofilin pathway and reduced smooth muscle contractility in prostate tissues.
Urethra − Urethral stricture	Urethral stricture resulted from excessive fibroblast proliferation, extracellular matrix deposition, and collagen synthesis in the urethral spongiosum—ROCK-dependent signaling pathway to facilitate scar formation. Treatment with fasudil (ROCK inhibitor) significantly suppressed fibroblast migration and reduced MLC, LIMK1, and cofilin expression.
Penis − Erectile dysfunction	Progressive fibrosis of cavernosal tissues after radical prostatectomy is the primary pathological mechanism of postoperative erectile dysfunction. The RhoA/ROCK1/LIMK2/cofilin pathway was involved in corporal fibrosis through coordination with TGF-β/sphingosine-1-phosphate signaling after cavernosal nerve injury. Short-term and long-term application of LX7101 (LIMK2 inhibitor) improved erectile function and restored cavernosal veno-occlusive dysfunction.

Note: LUTS/BPH, lower urinary tract symptoms/benign prostatic hyperplasia; tLMK2, testis-specific LIMK2 isoform; PAK, P21-activated kinase; PKC, protein kinase C; ROCK, Rho-associated protein kinase; MLC, myosin light chain.
